# Therapeutic Properties of Highly Selective β-blockers With or Without Additional Vasodilator Properties: Focus on Bisoprolol and Nebivolol in Patients With Cardiovascular Disease

**DOI:** 10.1007/s10557-021-07205-y

**Published:** 2021-06-09

**Authors:** Waleed AlHabeeb, Sanaa Mrabeti, Ahmed Adel Ibrahim Abdelsalam

**Affiliations:** 1 Cardiac Sciences Department, King Saud University, Riyadh, 12372 Saudi Arabia; 2 General Medicine and Endocrinology, Medical Affairs EMEA, Merck Serono Middle East FZ-LLC, Dubai, United Arab Emirates; 3 Cardiometabolic Care, GCC Medical Affairs, Merck Serono Middle East FZ-LLC, Riyadh, Saudi Arabia

**Keywords:** Beta blockade, Nebivolol, Bisoprolol

## Abstract

Bisoprolol and nebivolol are highly selective β_1_-adrenoceptor antagonists, with clinical indications in many countries within the management of heart failure with reduced left ventricular ejection fraction (HFrEF), ischaemic heart disease (IHD), and hypertension. Nebivolol has additional vasodilator actions, related to enhanced release of NO in the vascular wall. In principle, this additional mechanism compared with bisoprolol might lead to more potent vasodilatation, which in turn might influence the effectiveness of nebivolol in the management of HFrEF, IHD and hypertension. In this article, we review the therapeutic properties of bisoprolol and nebivolol, as representatives of “second generation” and “third generation” β-blockers, respectively. Although head-to-head trials are largely lacking, there is no clear indication from published studies of an additional effect of nebivolol on clinical outcomes in patients with HFrEF or the magnitude of reductions of BP in patients with hypertension.

## Introduction: differentiating between individual β-blockers

A variety of different therapeutic mechanisms exist within the β-blocker class. The prototype β-blocker, propranolol, and later agents such as bucindolol, carvedilol, labetolol, oxprenolol, nadolol, pindolol, sotalol, and timolol, do not demonstrate clinically significant differences in their selectivity for β_1_ and β_2_ adrenoreceptors [1–4]. By contrast, other β-blockers (e.g. acebutolol, atenolol, betaxolol, bisoprolol, celiprolol, esmolol, metoprolol, nebivolol, and xamoterol) are more or less selective for blockade of the β_1_ adrenoceptors found mainly in the heart over blockade of the β_2_ receptors that contribute to dilation of vascular and airway smooth muscle [1–4]. Some β-blockers (e.g. celiprolol, pindolol, acebutolol, and oxprenolol) induce some activation of β_1_-receptors (“intrinsic sympathomimetic activity” [ISA]), which tends to limit reductions in myocardial performance and heart rate during β-blockade, and reduces the potential for peripheral vasoconstriction, if directed against peripheral β_2_ adrenoreceptors [5]. The presence of ISA vs. no ISA does not appear to confer clinical benefit in patients with ischaemic heart disease (IHD) [6] or heart failure with reduced left ventricular ejection fraction (HFrEF) [7], however.

Other vasodilatory mechanisms expressed by individual β-blockers are additional blockade of α-adrenoceptors (bucindolol, carvedilol, celiprolol, labetalol) [1, 5, 8, 9], or promotion of release of nitric oxide (NO); by nebivolol, secondary to activation of β_3_-adrenoceptors nebivolol) [10–12]. These groups of β-blockers have been described as “first generation” (non-selective), “second generation” cardio/β_1_-selective, and “third generation” (with additional vasodilatory properties) [13].

### Purpose of this review

The differences between individual β-blockers, in terms of their mechanisms, selectivity and pharmacokinetic properties (among others) both facilitate to and complicate the design of individualised regimens for people with cardiovascular diseases [14, 15]. The purpose of our narrative review is to compare the therapeutic properties of a second generation and third generation cardioselective β-blocker in patients with hypertension, IHD and HFrEF. It is important to note that agents with lower β_1_-selectivity begin to block β_2_-adrenoceptors at higher doses (e.g. the European Summary of Product Characteristics for atenolol notes in this context that “selectivity decreases with increasing dose”). Accordingly, we have chosen two of the most highly selective β-blockers for this purpose, bisoprolol (second generation) and nebivolol (third generation).

### Methods

The review is based on literature searches for research articles with “bisoprolol” or “nebivolol” in the title. Priority is given to inclusion of randomised, active-controlled trials, given the extensive database of literature on β-blockers (placebo-controlled outcomes trials are included, as head-to-head comparative studies powered for clinical outcomes are lacking). References identified within these articles are also used in some cases. In each section, any available head-to-head comparisons between these agents will be reviewed first, followed by randomised, controlled comparisons of bisoprolol with nebivolol, as per above, with the structure of each subsection determined by the published evidence available. Other clinical trial evidence with bisoprolol or nebivolol is included at lower priority, where this sheds light on the overall therapeutic properties of these β-blockers.

## Properties of bisoprolol and nebivolol

### Relative selectivity for blockade of β_1_- vs. other adrenoceptors

Selectivity for the β_1_ adrenoreceptor has emerged as a key property within the β-blocker class, as described above and summarised in Table 1 [16–45]. Studies of the β_1_- vs. β_2_-adrenoceptor selectivity of bisoprolol and nebivolol have provided conflicting results. Nebivolol was found to be about threefold more selective than bisoprolol for the β_1_- vs. β_2_-adrenoceptor in human myocardium [46, 47], with similar findings reported from a study in humans which measured the effects of selective β-blockers on physiological responses to the β_2_-adrenoceptor agonist, terbutaline (both were more β_1_-selective than atenolol) [48]. However, other studies in human myocardium or cultured cells expressing human β_1_- or β_2_-adrenoceptorsfound that bisoprolol was more β_1_-selective than nebivolol [49]. An experimental study showed that bisoprolol and xamoterol were about 14-fold selective for the human β_1_- vs. the β_2_-adrenoceptor, compared with lower ratios for atenolol (~ fivefold), acebutolol and metoprolol (~ twofold) [50].

**Table 1:** Clinical significance of selective β_1_-adrenoceptor blockade

Body system	Implications of selective β_1_-adrenoceptor blockade
Peripheral vasoconstriction and PVD	Less blockade of peripheral β_2_-adrenoceptors with a selective agent reduces the likelihood feelings of cold in the extremities [16–18] Controlled clinical trials of bisoprolol (vs. lisinopril [19]) and nebivolol (vs. metoprolol [20]) have demonstrated effective BP lowering, and no cause for concern regarding worsening of limb ischaemia
Glycaemic control	Many reports have described a worsening of glycaemic control during treatment with a β-blocker and use of a cardioselective agent helps to minimise these effects [17, 18] The clinical significance of this phenomenon may have been overrated, however, worsened glycaemia may be unrelated to β-blockade [21],,and use of a β-blocker in a large diabetes prevention trial was not associated with increased risk of diabetes [22] Bisoprolol or nebivolol has not been associated with worsening of glycaemia [7, 23–29]
Asthma and COPD	Bronchospasm in patients with COPD or asthma may be exacerbated by blockade of β_2_-adrenoceptors in the smooth muscle of the airways [30] Non-selective β_1_-blockers, but not β_1_-selective agents, increase the risk of asthma exacerbations [31] A recent (2020) randomised, double-blind, crossover study confirmed that the bronchodilatory effects of bisoprolol were non-inferior during treatment with bisoprolol vs. placebo [32] Such findings have led to a reappraisal of the use of selective β_1_-blockers in patients with asthma or COPD [30, 33]; β_1_-selective agents are no contraindicated in Europe only for “severe bronchial asthma”
Erectile function	β-blockers, have been associated with new or exacerbated erectile dysfunction [34], although neither bisoprolol nor nebivolol were associated with sexual dysfunction [35–37] Nebivolol improved erectile function vs. metoprolol [38, 39], or atenolol (± chlorthalidone) [40] Another study demonstrated fewer patients reporting vs. not reporting sexual dysfunction on nebivolol vs. other β-blockers [41], or improved sexual function following a switch to nebivolol [42] This benefit for nebivolol may arise from its additional NO-releasing properties, a mechanism shared with the class of phosphodiesterase-5 inhibitors that are indicated for the management of male erectile dysfunction [43–45]

*COPD*, chronic obstructive airways disease; *NO*, nitric oxide; *PVD*, peripheral vascular disease

The heart also contains β_3_-adrenoceptors. Binding studies showed that bisoprolol was 31-fold selective for blockade of β_1_-adrenoceptors vs. β_3_-adrenoceptors [51]. Nebivolol is a partial agonist at β_3_-receptors (see below).

### Intrinsic sympathomimetic activity

A number of β-blockers demonstrate intrinsic sympathomimetic activity, i.e. they have partial agonist activity at different β-adrenoceptors [52–55]. Neither bisoprolol nor nebivolol has intrinsic sympathomimetic activity at the β_1_- or β_2_-adrenoceptor [52–55].

Nebivolol (but not bisoprolol) exerts intrinsic sympathomimetic activity at the β_3_-adrenoceptor, which is associated with a vasodilator action secondary to increased production of nitric oxide [12, 56, 57]. This action may account for observations of a lesser effect of nebivolol in reducing heart rate, compared with bisoprolol (see below) [51, 55, 56, 58].

### Inverse agonism at the β_1_-adrenoceptor

Constitutive activity (some level of activation of a receptor in the absence of its specific agonist) has been demonstrated for β_1_-adrenoceptors [59]. An inverse β_1_ agonist can suppress the level of activation of a receptor to a level below that seen in the absence of an agonist [59]. Both bisoprolol and nebivolol are inverse agonists at the β_1_- receptor (as are most β_1_-adrenoceptor blockers [49, 56]); however, the level of inverse agonism of these drugs appears to be similar and therefore unlikely to contribute to differences in their therapeutic actions [49]. The phenomenon of inverse agonism has been associated with prevention of desensitisation of receptors, and thus with increased numbers of β_1_-adrenoceptors [56]. While this is of theoretical benefit in the setting of HFrEF, variations in the ability of different β-blockers to upregulate β_1_-adrenoceptor numbers have not been associated with differences in their beneficial effects on cardiac performance [56].

### Other sympatholytic actions

β-blockers are sympatholytic, in that they inhibit the actions of the sympathetic neurotransmitters at the level of the β-adrenoceptors [51–55]. Additional mechanisms may be at play, including blockade of α-adrenoceptors (labetalol, carvedilol), modulation of baroreflex function, effects on presynaptic β_1_-adrenoceptors that modulate sympathetic neurotransmitter release, or actions to limit sympathetic outflow from the brain (for lipophilic agents that cross the blood–brain barrier) [52–55].

### Pharmacokinetics

Table 2 summarises the pharmacokinetics of bisoprolol and nebivolol [12, 60–63]. Both drugs have elimination half life (T½) consistent with once-daily dosing, and are absorbed relatively rapidly. Nebivolol, but not bisoprolol, is subject to an extensive, but variable, first pass metabolism, with potential for pharmacokinetic interactions with other inhibitors of CYP450 2D6 noted in the European labelling for nebivolol. Differences in the rate of metabolism of nebivolol between individuals affect the pharmacokinetics of nebivolol, with longer time to maximal plasma concentration (Tmax) and a longer T½. In addition, there is a ~ eightfold difference in bioavailability and ~ 15-fold difference in exposure to nebivolol between extensive and poor metabolisers of the drug, and the mode of elimination differs according to metaboliser phenotype. The level of overall β_1_-blockade is similar between these metaboliser phenotypes following a dose of nebivolol; however, due to the greater presence of active metabolites of nebivolol in extensive metabolisers. Nebivolol is almost completely protein bound, compared with only about 30% for bisoprolol.

**Table 2:** Overview of the pharmacokinetic properties of bisoporol and nebivolol

	Bisoprolol	Nebivolol
T_½_ (h)	10–12	~ 12 ha
T_max_ (h)	1.5–5	1.5–4 h^b^
First-pass metabolism	Low (~ 10%)	Extensive (CYP450 2D6)
Mode of elimination	50% unchanged in urine, 50% hepatic (metabolites excreted in urine)	35% via urine, 44% via faeces^c^
Plasma protein binding	30%	98%
Active metabolites?	No	Yes
Absorption affected by food?	No	No

Figures shown are average metabolisers and are ^a^19 h, b3–6 h, ^c^67% urine 13% faeces, in poor metabolisers of the drug. See text for references

Variations in renal function have been observed to contribute to variability of plasma concentrations of orally-administered bisoprolol in a population with diabetes, and it may be useful to measure renal function to support individualised dosing with bisoprolol [64]. Another study analysed 31 covariates and found that only higher bisoprolol doses and cigarette smoking (both of which were postulated to induce enzymes in the cytochrome P450 system) influenced the PK of bisoprolol [65].

The pharmacokinetic profile of nebivolol is therefore complex, compared with bisoprolol. A recent study showed that exposure to nebivolol (and metoprolol, propranolol, and carvedilol) in pharmacokinetic studies was more variable than exposure to bisoprolol (and atenolol, sotalol, labetalol, nadolol, and pindolol), based on the coefficient of variation of the area under the plasma-concentration–time curve after oral dosing of these drugs [66].

Genome-wide association studies (GWAS) have demonstrated an association between effects on blood pressure of β-blockers in general and single nucleotide polymorphisms (SNPs) in the *BST1* or *PTPRD* gene, although there was no suggestion of differences between the antihypertensive efficacy of individual β-blockers according to the presence or absence of this mutation [67, 68]. It has also been suggested that common variations in the enzyme CYP2D6 may alter the efficacy of safety of metoprolol to a significant extent in patients in certain geographical regions. This may be expected to hold true also for other β-blockers subjected to extensive metabolism by the cytochrome P450 system [69]. One GWAS identified SNPs in three genes that appeared to modulate the antihypertensive response to bisoprolol but not to atenolol, although the functional significance of this is unclear at present [70]. Elsewhere, a SNP (Arg189Gly) influenced heart rate responses to carvedilol, but not to bisoprolol [71].

## Bisoprolol and nebivolol in heart failure

### Randomised head-to-head comparisons and meta-analyses

A randomised crossover study evaluated 2 months treatment with each of carvedilol, bisoprolol and nebivolol in 61 patients with HFrEF of moderate severity [72]. There were some differences between groups in oxygen perfusion and response to hypoxia, in which the authors considered could contribute to individualised patient care, but there were no significant differences between treatments for NYHA classification, Minnesota heart failure questionnaire scores, renal function, levels of B-type natriuretic peptide, echocardiography findings, or lung mechanics. One small study showed that treatment for 2 weeks with nebivolol increased left ventricular function in comparison with bisoprolol, celiprolol or carvedilol, in healthy volunteers, apparently associated with improved diastolic function and myocardial compliance, although the relevance of this study to patients with HFrEF is unclear [73].

**Fig. 1:**
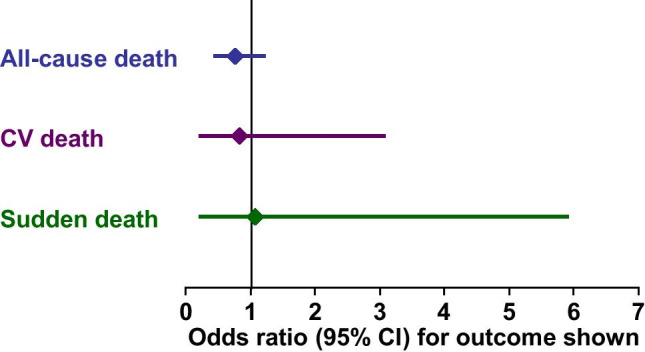
Comparison of the effects of bisoprolol and nebivolol on mortality outcomes from a network meta-analysis. Odds ratios < 1 favour bisoprolol. Drawn from data presented in reference [74]

A network meta-analysis of outcomes trials in HFrEF [74]included the CIBIS trials with bisoprolol [75–77] and the SENIORS trial with nebivolol [78] (see below for more details of these trials). There was no significant difference between bisoprolol and nebivolol for effects on overall mortality, cardiovascular mortality, or sudden death (Fig. 1).

### Other randomised outcomes trials

No randomised, head-to-head outcomes trials have compared bisoprolol and nebivolol in patients with HFrEF. Accordingly, this section reviews briefly the outcomes trials that have been conducted separately with these agents.

Three randomised outcomes trials [75–77] have evaluated bisoprolol in comparison with placebo (each plus standard of care) in patients with HFrEF (Table 3). The first of these, the Cardiac Insufficiency Bisoprolol Study (CIBIS) trial demonstrated symptomatic improvement in patients randomised to bisoprolol vs. placebo, but the small patient population precluded demonstration of improved clinical outcomes [75]. CIBIS II recruited a larger population, and this trial concluded prematurely following the emergence of a significant reduction in mortality (the primary outcome of the trial) in the bisoprolol vs. placebo group [76]. Post-hoc analyses from these trials showed that outcome benefits were similar whether or not patients had diabetes, renal impairment, or NYHA class IV symptoms, were elderly, or were also receiving digitalis, amiodarone or aldosterone antagonists [79].

**Table 3:** Randomised outcomes trials that evaluated bisoprolol or nebivolol in patients with congestive heart failure

Trial	N, duration	Patients	Comparator	Primary endpoint	Main findings
Bisoprolol					
CIBIS [75]	641, 1.9 y	NYHA class III–IV LVEF < 40%	Placebo (+ usual care^a^)	All-cause mortality	Fewer hospitalisations for HF on bisoprolol (61) vs. placebo (90) (p < 0.01) More improved ≥ 1 NYHA class (48 on placebo vs. 68 on bisoprolol, p = 0.04) No differences for mortality (RR [0.56 to 1.15], p = 0.22)
CIBIS II [76]	2,647, Mean 1.3 y	NHYA class III–IV LVEF ≤ 35	Placebo (+ usual care^b^)	All cause mortality	Significant mortality benefit for bisoprolol (HR 0.66 [0.54 to 0.81], p < 0.0001) led to premature conclusion of the trial Fewer sudden deaths on bisoprolol vs. placebo (HR 0.56 [0.39 to 0.80], p = 0.0011)
CIBIS III [77]	1,010, Up to 2.5 y^c^	Mild-to-moderate HFrEF LVEF ≤ 35	Enalapril^c^	All-cause mortality or hospitalisation	No significant difference between initial treatment with bisoprolol or enalapril on the primary endpoint (HR 0.94 [0.77 to 1.16])
Nebivolol					
SENIORS [78]	2,128 Mean 1.75 y	Age ≥ 70 y LVEF ≤ 35	Placebo	All-cause mortality or cardiovascular hospital admission	Risk of primary endpoint of all-cause death or hospitalisation for cardiovascular cause reduced for nebivolol vs. placebo (HR 0.86 [0.74 to 0.99], p = 0.039) No significant effect on all-cause mortality (HR 0.88 [0.71 to 1.08], p = 0.21)

Usual care = ^a^diuretic + vasodilator (90% were on ACEI); ^b^diuretics + ACE inhibitor. ^c^In CIBIS III, patients received randomised monotherapy for 6 months followed by bisoprolol + enalapril in combination for 6–24 months. *HF*, heart failure; *LVEF*, left ventricular ejection fraction; *NYHA*, New York Heart Association. Numbers in square brackets are 95%CI

The third trial, CIBIS III, was designed to evaluate whether starting therapy with bisoprolol and then adding enalapril was superior to starting with enalapril and subsequently adding bisoprolol [77]. There were no differences in clinical outcomes between either method of achieving this evidence-based combination. However, a post-hoc analysis of CIBIS III showed that titration of either therapy to at least half-maximal dose was more likely for the treatment that was prescribed first, suggesting that education of physicians on optimisation of heart failure therapies is required [80].

The Study of the Effects of Nebivolol Intervention on Outcomes and Rehospitalization in Seniors with Heart Failure (SENIORS) trial evaluated nebivolol in an elderly population with heart failure (Table 3) [78, 81]. Randomisation to nebivolol vs. placebo for an average of 21 months significantly reduced the risk of the primary composite endpoint (all cause mortality or cardiovascular hospital admission) vs. placebo, and this effect was independent of gender, age or LVEF. There was no significant effect on all-cause mortality, a secondary endpoint, however. Sub-analyses from SENIORS showed that nebivolol improved clinical outcomes similarly in patients with and without moderate renal dysfunction [81], or low SBP at baseline, a risk factor for adverse clinical outcomes in this population [82].

## Bisoprolol and nebivolol in ischaemic heart disease

### Randomised outcomes trials

No outcome trial has compared bisoprolol with nebivolol directly in patients with IHD. The Total Ischemic Burden Bisoprolol Study randomised 330 patients with CHD (defined as stable angina, a positive exercise test and at ≥ 2 transient episodes of myocardial ischaemia during 48 h of ambulatory ECG recording) to bisoprolol or nifedipine [83, 84]. Bisoprolol reduced the number of episodes of ischaemia vs. nifedipine at 4 weeks. Follow-up at one year revealed a lower rate of a composite of major adverse cardiac events (MACE; cardiac and non-cardiac death, nonfatal acute MI, hospital admission for unstable angina, or revascularisation) in the bisoprolol (22%) vs. nifedipine groups (22 vs. 33%, p = 0.03).

### Other randomised trials

A randomised trial found that treatment with bisoprolol was associated with improved survival of myocardium (assessed using ^99^Tcm-sestamibi uptake) in patients with angina pectoris [85]. The efficacy of bisoprolol in managing anginal attacks has been shown to be comparable to that of atenolol [86–90] or verapamil [91] in randomised trials.

ECG ST segment elevation was significantly lower after randomisation to nebivolol vs. atenolol, in patients with documented CHD in patients undergoing exercise testing within a randomised trial [92]. A one-year randomised trial evaluated nebivolol in comparison with metoprolol and carvedilol in 172 patients with acute MI complicated by left ventricular dysfunction [93]. A composite outcome of nonfatal MI, cardiovascular death, hospitalisation for unstable angina pectoris or heart failure, stroke or revascularisation was significantly lower in the nebivolol vs. metoprolol groups (14.5 vs. 31.5%; p = 0.03), with no significant (p > 0.05) trends for benefit with for nebivolol vs. carvedilol (14.5 vs. 20.3%) and carvedilol vs. metoprolol (20.3 vs. 31.5%, p > 0.05).

## Bisoprolol and nebivolol in hypertension

### Head-to-head comparisons and meta-analyses

Similar magnitudes of blood pressure (BP) lowering were observed in patients (N = 273) randomised to bisoprolol (mean change –16/–7 mmHg) or nebivolol (mean change –16/–6 mmHg) for 12 weeks in a randomised, single-blind trial (the NEBIS study) [59]. Another small, randomised, crossover trial compared the effects of nebivolol and bisoprolol on endothelial function in 25 patients with hypertension [94]. Effects on endothelial function (forearm flow-mediated vasodilatation) were larger in the nebivolol group, consistent with its additional mechanism of enhanced synthesis of NO in the vasculature (see above). This did not translate into a statistically significant difference between groups for effects on BP, however: mean BP was reduced from 152/99 mmHg at baseline to 132/82 mmHg for nebivolol and to 130/83 mmHg for bisoprolol. Clinical outcomes after one year of treatment were evaluated in another head-to-head comparison of bisoprolol and nebivolol, conducted in 1056 patients with hypertension [95]. Randomisation to nebivolol vs. bisoprolol was associated with small and statistically non-significant differences between groups for overall mortality (9.8 vs. 11.5%), cardiovascular mortality (5.4 vs. 7.0%), hospitalisation for any cause (14.4 vs. 16.3%), and hospitalization cardiovascular for a cardiovascular cause (9.8 vs. 12.1%).

A meta-analysis showed that the proportion of patients achieving BP targets was similar for nebivolol and other β-blockers [49]. Statistically significantly higher percentages of patients achieving these targets were observed for nebivolol vs. ACE inhibitors and ARBs in this analysis [96].

Reductions in the resting heart rate are an important determinant of improved prognosis in patients at risk of adverse cardiovascular outcomes who receive cardioselective β-blockade [97]. As above, head-to-head data on the effects of these β-blockers on heart rate is lacking. However, there was a strong trend (p = 0.06) towards a larger decrease in heart rate 3 h after administration with bisoprolol (mean change –24 bpm) vs. nebivolol (mean change –15 bpm) in the randomised NEBIS study [58].

Limitations of these head-to-head comparisons in patients with hypertension included a relatively short duration (16 weeks or less [58, 93], or small patient populations (25 or less) [94]. The randomised outcomes trial that compared these agents was conducted in a single centre, with a short follow-up duration (one year), and differences in BMI at baseline between groups may have confounded outcomes to some extent [95]. Longer, better powered, head-to-head comparisons of bisoprolol and nebivolol are required in patients with hypertension.

### Other randomised, active-controlled trials

An overview of the results of randomised, active-controlled evaluations of bisoprolol and nebivolol given as monotherapy to patients with hypertension is shown in Table 4 [20, 23, 98–135]. In general, the efficacy on BP of these drugs was similar to comparators, except for a greater effect on BP (especially 24-h BP) with bisoprolol vs. atenolol.

**Table 4:** Overview of efficacy on blood pressure (BP) of monotherapy with bisoprolol or nebivolol in randomised, active-controlled trials

Comparator	Bisoprolol trials	Nebivolol trials
β-blockers		
Acebutolol	Similar in hypertension [98]	–
Atenolol	Similar in hypertension [99–103] **(lower central BP on bisoprolol** in one study [99]) **More BP lowering with bisoprolol** in elderly, and non-Black hypertensive patients (similar effects in younger and Black patients) [102] In mild-to-moderate hypertension: Similar effects between treatments, [104] or… Similar effects on office BP but **greater effect of bisoprolol on 24 h BP** [104] ** Greater effect of bisoprolol on BP** [105, 106] Similar effects on sitting BP but **greater effect on standing BP with bisoprolol** [107]	Similar in essential hypertension [98, 123, 124] Similar in isolated systolic hypertension [124] Similar in hypertension + type 2 diabetes [125] Similar in hypertensive patients undergoing isometric stress [126]
Carvedilol	–	Similar in mild-to-moderate primary hypertension [127]
Celiprolol	Similar in hypertension: **lower central BP on celiprolol** [108]	–
Metoprolol	Similar in mild-to-moderate hypertension [109] **Greater effect of bisoprolol on BP during exercise in hypertension** [110]	Similar in hypertension + intermittent claudication [20]
RAAS blockers		
Captopril	Similar in elderly patients with hypertension [111]	–
Lisinopril	Similar in hypertension (effects on ambulatory BP) [112] Similar in mild-to-moderate hypertension [113]	Similar in hypertension [130]
Enalapril	Similar in hypertension (office and 24 h BP) [114] Similar in mild-to-moderate hypertension [115]	Similar in hypertension [131, 132]
Irbesartan	–	Similar in isolated systolic hypertension [128]^a,b^
Losartan	Similar effects on brachial BP, larger effect of losartan on central BP [116]^b^ Similar in recently diagnosed hypertension [117]	–
Valsartan	–	Similar in hypertension and obstructive sleep apnoea [129]
Calcium channel blockers		
Amlodipine	–	Similar in elderly hypertensive patients [133]
SR nifedipine	Similar in mild to moderate hypertension [115] Similar in hypertensive elderly patients [118, 119]	Similar in hypertension [134, 135]
Diuretics		
Chlorthalidone	Similar in arterial hypertension [120]	–
Spironolactone	**Spirololactone more effective** in drug-resistant hypertension [121]^c^	–

^a^Effects on 24 h BP. ^b^Each drug in combination with hydrochlorothiazide. ^c^High blood pressure despite prior treatment with a renin–aldosterone system (RAAS) blocker, a calcium channel blocker (CCB) and a diuretic. SR: sustained release. A head-to-head randomised comparison of bisoprolol with nebivolol in patients with hypertension is not shown here (see text and reference [58])

A meta-analysis showed that nebivolol reduces BP effectively across age groups, though with slightly less efficacy in older patients [20]. Nebivolol was also shown to be effective in African-American patients with hypertension, who have been identified as a population who respond less to beta-blockade than people of other ethnicities [136]. Clinical pharmacology studies showed that nebivolol improved the function of large [124, 137] or small [138] arteries more than atenolol, despite a similar overall effect on BP, consistent with the results of the clinical comparison with bisoprolol, described above [58]. Such an effect was not seen in a randomised comparison with metoprolol, however [139]. Another mechanistic study showed that nebivolol, but not metoprolol, reduced BP in hypertensive patients with autonomic failure, a condition that is known to respond to NO [140].

Nebivolol and bisoprolol are effective in combination with other antihypertensive agents for the management of hypertension, consistent with other drugs used for this purpose [141–146]. A full account of such studies is beyond the scope of this review.

## Discussion

### How do these second- and third-generation β-blockers compare?

The use of both bisoprolol and nebivolol for the management of HFrEF is supported by the results of randomised, placebo-controlled outcomes trials (the CIBIS programme for bisoprolol and the SENIORS trial for nebivolol). We have no head-to-head outcomes trials between these drugs in this (or any other) indication, although one such trial revealed little difference between them in terms of effects on clinically important and commonly measured parameters relevant to heart failure [72]. A network meta-analysis of the CIBIS programme and SENIORS did not reveal any sign of a clinically or statistically significant difference in heart failure outcomes between these agents (see Fig. 1) [76].

β-blockade is firmly established within the management of HFrEF, and both bisoprolol and nebivolol (along with carvedilol and metoprolol) are identified as evidence-based treatments in major European guidelines for HFrEF management [147], while bisoprolol is among medications “commonly used for HFrEF” in US guidelines [148]. Similarly, β-blockade is recommended as a first-line therapy option for chronic IHD and following an acute coronary syndrome in Europe [149–151], and in the USA [152]. No individual β-blocker is specified in these guidelines, so that the efficacy of β-blockade is essentially regarded as a class effect. Similarly, guidelines for the management of hypertension note that β-blockers in general have been shown to reduce the risk of MACE [153], consistent with the conclusion from a large meta-analysis that the magnitude of BP reduction per se may be the main driving force in reducing the risk of MACE [154]. The European guidelines point out that neither bisoprolol nor nebivolol is currently supported by a cardiovascular outcomes trials [151].

Finally, we have concentrated on evidence from randomised outcomes trials wherever possible in this article, and have not addressed real-world evidence, which is increasingly influential in the evaluation o the effects of therapies in routine care. One real world analysis conducted in more than 7500 patients with hypertension treated for up to 14 years, found that treatment with bisoprolol was associated with reduced mortality compared with pooled data for other β-blockers (HR 0.45 [95%CI 0.34 to 0.61) or pooled non-β-blocker therapies (HR 0.50 [95%CI: 0.38 to 0.66]) [155]. A fuller consideration of real-world evidence in this area would be an interesting subject for review elsewhere.

### Knowledge gaps and outstanding research questions

The only reliable comparison of the effects of two drugs on a given disease is within a head-to-head randomised clinical trial. We have only one small trial comparing bisoprolol and nebivolol in HFrEF [72], and no such trials in patients with IHD or hypertension. The evidence base for improved clinical outcomes with bisoprolol and nebivolol is strongest in patients with HFrEF, and more information is required on the effects of these outcomes in patients with IHD and, especially, hypertension. In addition, the possible place of β-blockade in heart failure with preserved ejection fraction remains controversial: data to date from trials of β-blockers in these patients have been inconsistent [156], although a recent registry study suggested benefit from this approach [157]. Further studies in these patients are required.

## Conclusions

A high level of β_1_-adrenoceptor selectivity is contributes importantly to the utility of β-blockers in the management of hypertension and cardiovascular disease. This will be especially so outside the tightly controlled environments of clinical trials, where patients may have (or develop over time) comorbidities such as dysglycaemia, obstructive pulmonary disease or erective dysfunction. Bisoprolol and nebivolol are both highly cardioselective β-blockers, rendering them suitable for use in many patients with such comorbidities, compared with non-selective (first-generation) β-blockers. Nebivolol, a third-generation β-blocker, has additional vasodilator actions, related to enhanced release of NO in the vascular wall, compared with bisoprolol, a second-generation agent. The additional NO-releasing effect of nebivolol may underlie reports of improvements in erectile function in patients receiving this agent. Available evidence, summarised in this review, does not provide evidence of either a superior effect on clinical outcomes in HFrEF, or a greater effect on BP in hypertension, for nebivolol vs. bisoprolol, although head-to-head trials are lacking.

## Data Availability

Not applicable.
